# Supplementary material for: Transient deSUMOylation of IRF2BP proteins controls early transcription in EGFR signaling
Source: EMBO Rep. 2021 Jan 22;22(3):e49651. doi: 10.15252/embr.201949651 (PMC7926235; doi:10.15252/embr.201949651)
Supplement: Supplementary file 1 — Expanded View Figures PDF [file EMBR-22-e49651-s001.pdf]

# Expanded View Figures

A

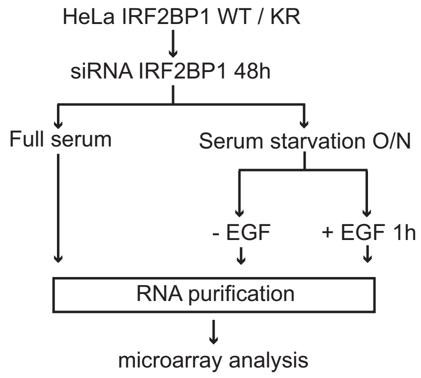


B

GO analysis (Top 10) of differentially expressed genes (1.5 fold) in K579R vs WT in the “full serum” condition

| GO biological process complete | Fold enrichm. | -log10 (FDR) |
|---|---|---|
| biological adhesion | 5.06 | 6.79 |
| cell adhesion | 4.90 | 6.55 |
| negative regulation of cell proliferation | 5.72 | 6.38 |
| regulation of multicellular organismal process | 2.60 | 5.36 |
| regulation of cell proliferation | 3.43 | 5.36 |
| regulation of cell. resp. to growth factor stim. | 8.16 | 4.10 |
| anatomical structure morphogenesis | 2.83 | 4.10 |
| regulation of cell adhesion | 4.90 | 4.09 |
| multicellular organism development | 2.00 | 4.08 |
| regulation of developmental process | 2.57 | 3.99 |

C

GO analysis (Top 10) of differentially expressed genes (1.5 fold) in K579R vs WT in the “-EGF” condition

| GO biological process complete | Fold enrichm. | -log10 (FDR) |
|---|---|---|
| regulation of cell proliferation | 3.61 | 4.51 |
| negative regulation of cell proliferation | 5.46 | 4.32 |
| negative regulation of signaling | 3.48 | 2.96 |
| negative regulation of cell communication | 3.49 | 2.86 |
| tube development | 4.30 | 2.83 |
| system development | 2.07 | 2.67 |
| multicellular organism development | 1.95 | 2.59 |
| regulation of protein ser/thr kinase activity | 5.25 | 2.54 |
| cell migration | 3.83 | 2.54 |
| biological adhesion | 3.85 | 2.53 |

D

GO analysis (Top 10) of differentially expressed genes (1.5 fold) in K579R vs WT in the “+EGF” condition

| GO biological process complete | Fold enrichm. | -log10 (FDR) |
|---|---|---|
| biological adhesion | 4.03 | 2.99 |
| cell adhesion | 4.05 | 2.73 |
| regulation of smooth muscle cell migration | 17.55 | 2.35 |
| anatomical structure morphogenesis | 2.57 | 2.35 |
| negative regulation of cell proliferation | 4.31 | 2.34 |
| extracellular structure organization | 6.03 | 2.28 |
| regulation of cell proliferation | 2.86 | 2.27 |
| positive reg. of cellular component movem. | 4.77 | 2.12 |
| multicellular organism development | 1.84 | 2.11 |
| developmental process | 1.75 | 2.00 |

**Figure EV1. Transcriptome analyses of HeLa cells expressing either wt IRF2BP1 or the SUMOylation-deficient variant IRF2BP1 K579R.**

A Stable IRF2BP1 cell lines were used to perform microarray analyses in asynchronously growing cells (full serum) and under serum starvation, with or without EGF treatment for 1 h.

B–D GO analysis revealed that differentially expressed genes cluster in similar biological processes, such as adhesion, proliferation, and signaling processes, irrespective of whether they were grown in full serum (B), under serum starvation (C), or under serum starvation and EGF treatment (100 ng/ml) for 1 h (D).

© 2021 The Authors

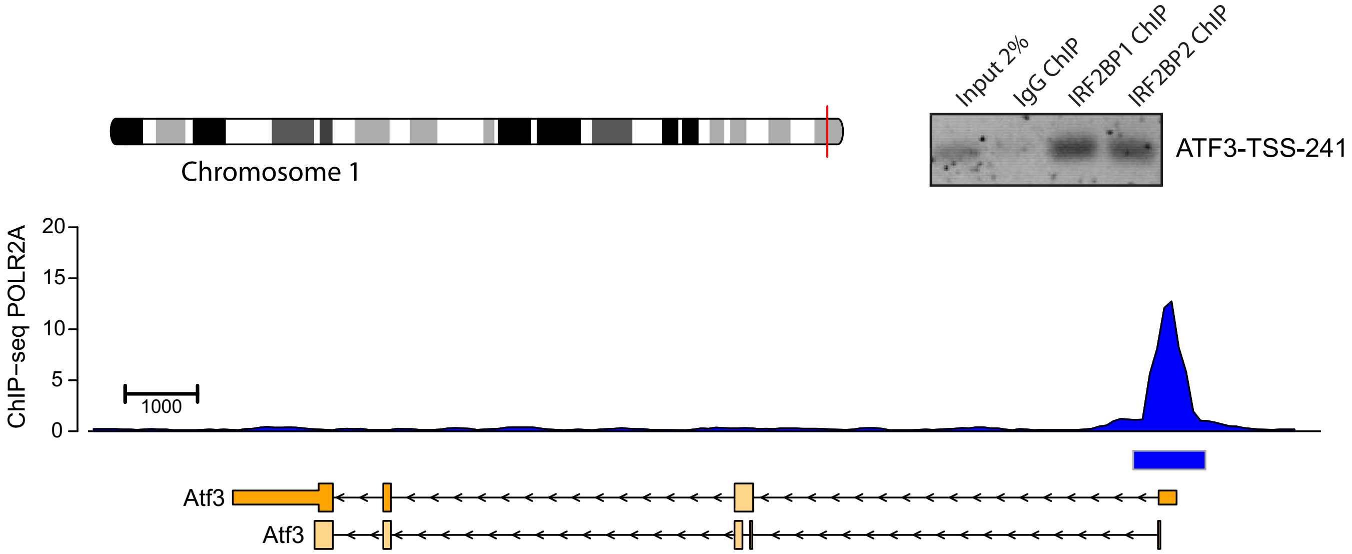


**Figure EV2. Chromatin IP reveals association of human IRF2BP1 and IRF2BP2 with the proximal ATF3 promoter in HeLa cells.**

Gene architecture of human ATF3. The primers at -358/-241 ("ATF3-TSS-241") relative to the TSS were used for ChIP experiments.

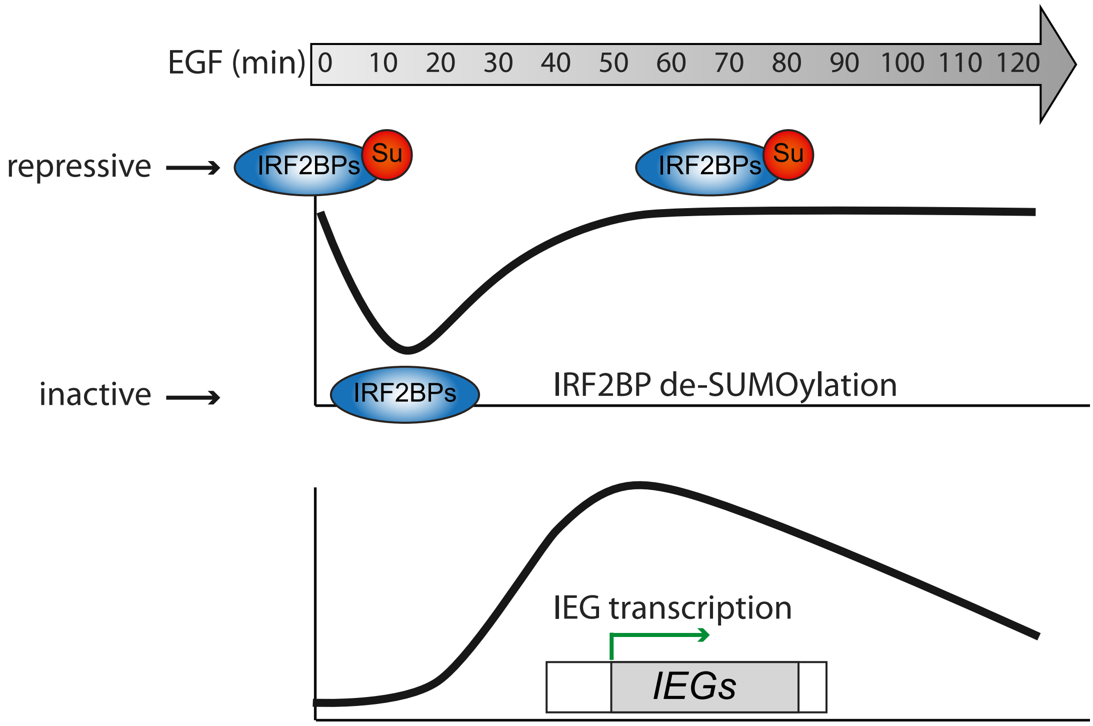


**Figure EV3. Model.**

DeSUMOylation of IRF2BP1 occurs within 15 min after EGF treatment. This transient event directly correlates with the gene expression of immediate early genes.


© 2021 The Authors

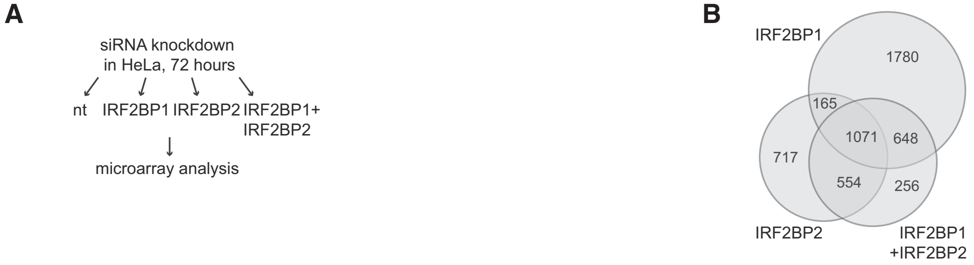


**C**

GSEA analysis of siRNA IRB2BP1 microarray (KEGG dataset, Top 15)
ranked by Normalized Enrichment Score (NES)

| NAME | NES | FDR |
|---|---|---|
| KEGG_ADHERENS_JUNCTION | 2.244 | 5.77E-04 |
| KEGG_BASAL_CELL_CARCINOMA | 2.159 | 6.67E-04 |
| KEGG_PROSTATE_CANCER | 2.055 | 1.76E-03 |
| KEGG_MELANOGENESIS | 2.025 | 2.19E-03 |
| KEGG_RENAL_CELL_CARCINOMA | 1.981 | 3.39E-03 |
| KEGG_TGF_BETA_SIGNALING_PATHWAY | 1.913 | 6.55E-03 |
| KEGG_HEDGEHOG_SIGNALING_PATHWAY | 1.880 | 8.93E-03 |
| KEGG_COLORECTAL_CANCER | 1.864 | 9.36E-03 |
| KEGG_PANCREATIC_CANCER | 1.861 | 8.75E-03 |
| KEGG_ERBB_SIGNALING_PATHWAY | 1.854 | 8.45E-03 |
| KEGG_FC_GAMMA_R_MEDIATED_PHAGOCYTOSIS | 1.816 | 1.18E-02 |
| KEGG_GNRH_SIGNALING_PATHWAY | 1.798 | 1.37E-02 |
| KEGG_GLYCOSAMINOGLYCAN_BIOSYNTHESIS_CHONDROITIN_SULF | 1.793 | 1.34E-02 |
| KEGG_WNT_SIGNALING_PATHWAY | 1.789 | 1.30E-02 |
| KEGG_ENDOMETRIAL_CANCER | 1.788 | 1.23E-02 |

**D**

GSEA analysis of siRNA IRB2BP2 microarray (KEGG dataset, Top 15)
ranked by Normalized Enrichment Score (NES)

| NAME | NES | FDR |
|---|---|---|
| KEGG_MTOR_SIGNALING_PATHWAY | 1.896 | 5.78E-02 |
| KEGG_FC_GAMMA_R_MEDIATED_PHAGOCYTOSIS | 1.778 | 1.23E-01 |
| KEGG_GLYCOSAMINOGLYCAN_BIOSYNTHESIS_CHONDROITIN_SULF | 1.754 | 1.09E-01 |
| KEGG_JAK_STAT_SIGNALING_PATHWAY | 1.724 | 1.06E-01 |
| KEGG_O_GLYCAN_BIOSYNTHESIS | 1.717 | 8.92E-02 |
| KEGG_ENDOMETRIAL_CANCER | 1.701 | 8.66E-02 |
| KEGG_TOLL_LIKE_RECEPTOR_SIGNALING_PATHWAY | 1.689 | 8.23E-02 |
| KEGG_CHRONIC_MYELOID_LEUKEMIA | 1.685 | 7.57E-02 |
| KEGG_ACUTE_MYELOID_LEUKEMIA | 1.646 | 9.72E-02 |
| KEGG_GLYCEROPHOSPHOLIPID_METABOLISM | 1.606 | 1.25E-01 |
| KEGG_APOPTOSIS | 1.604 | 1.16E-01 |
| KEGG_ERBB_SIGNALING_PATHWAY | 1.601 | 1.10E-01 |
| KEGG_PHOSPHATIDYLINOSITOL_SIGNALING_SYSTEM | 1.578 | 1.24E-01 |
| KEGG_PANCREATIC_CANCER | 1.577 | 1.16E-01 |
| KEGG_VEGF_SIGNALING_PATHWAY | 1.575 | 1.09E-01 |

**Figure EV4. Transcriptome analyses of Hela cells with or without depletion of IRF2BP1, IRF2BP2, or both proteins.**

A Schematic representation of the microarray experiment that was performed in this study (three independent experiments): IRF2BP1, IRF2BP2, and IRF2BP1 + IRF2BP2 were knocked down in HeLa cells for 72 h, and gene expression data were recorded by microarray; non-targeting siRNAs were used as a control.

B Venn diagram of genes that are differentially expressed (at least 1.5-fold, FDR < 0.05) upon IRF2BP-knockdown compared to control. A large number of genes are dependent on IRF2BP proteins, and they can be overlapping or distinct for IRF2BP1 and IRF2BP2.

C, D Gene set enrichment analyses (GSEA) of the microarray data using KEGG gene set. Many signaling pathways were enriched upon knockdown of IRF2BP1 (C) and IRF2BP2 (D). The first 15 hits ranked by Normalized Enrichment Score (NES) are shown.
