# Supplementary material for: Transient deSUMOylation of IRF2BP proteins controls early transcription in EGFR signaling
Source: EMBO Rep. 2021 Jan 22;22(3):e49651. doi: 10.15252/embr.201949651 (PMC7926235; doi:10.15252/embr.201949651)
Supplement: Supplementary file 2 — Table EV1 [file EMBR-22-e49651-s002.docx]

**Table EV1:** Key Resource Table. All resources and reagents (with catalogue and lot numbers, where applicable), including antibodies, bacterial strains, chemicals, peptides, recombinant proteins, critical commercial assays, deposited data, cell lines, oligonucleotices, recombinant DNAs and software used in this study are listed.

| **REAGENT or RESOURCE** | **SOURCE** | **IDENTIFIER** |
| --- | --- | --- |
| Antibodies | | |
| Rabbit polyclonal anti-IRF2BP1 | Proteintech Group | 13698-1-AP  varying Lots |
| Rabbit polyclonal anti-IRF2BP2 | Proteintech Group | 18847-1-AP  varying Lots |
| Rabbit polyclonal anti-TRIM24 | Proteintech Group | 14208-1-AP  varying Lots |
| Rabbit polyclonal anti-Ubc9 (H-81) | Santa Cruz Biotechnology | Sc-10759  Lot # D1411 |
| Goat polyclonal anti-RanGAP | (Hutten *et al*, 2008) |  |
| Goat polyclonal anti-Uba2 | (Bossis & Melchior, 2006) |  |
| Mouse monoclonal anti-HA.11 (clone 16B12) | Covance | MMS-101P-500  Lot # B204540 |
| Rabbit polyclonal anti-GFP (FL) | Santa Cruz Biotechnology | sc-8334,  Lot # C2614 |
| Rabbit polyclonal anti-TIF1beta (=TRIM28) | Cell Signaling Technology | 4124  Lot # 1 |
| Mouse monoclonal anti-beta-actin (clone AC-15) | Sigma-Aldrich | A1978 |
| Rabbit polyclonal anti-MKP-1 (M-18) (=Dusp1) | Santa Cruz Biotechnology | sc-1102,  Lot # F2211 |
| Rabbit polyclonal anti-DUSP1/MKP1 (E8L7D) | Cell Signaling Technology | 48625S  Lot # 1 |
| Mouse monoclonal anti-SUMO1 (21C7) | This paper (purified from hybridoma cells) | SUMO-1 21C7-s |
| Mouse monoclonal anti-SUMO2/3 (8A2) | This paper (purified from hybridoma cells) | SUMO-2 8A2-s |
| Rabbit polyclonal anti-SUMO-2+3 | Eurogentec | AV-SM23-100 |
| Rabbit monoclonal anti-SUMO2/3 (18H8) | Cell Signaling Technology | 4971S  Lot # 3 |
| Goat polyclonal anti-mouse-HRP | Jackson Immuno Research | 715-035-150 |
| Goat polyclonal anti-rabbit-HRP | Jackson Immuno Research | 711-035-152 |
| Goat polyclonal anti-goat-HRP | Jackson Immuno Research | 705-035-147 |
| Bacterial Strains | | |
| NEB 5-alpha competent *E. coli* (High Efficiency) | New England Biolabs | C2987H  varying Lots |
| Chemicals, Peptides, and Recombinant Proteins | | |
| Human EGF | Peprotec | AF-100-15  varying Lots |
| SUMO1-epitope peptide  (VPMNSLRFLFE) | Peptide synthesis by DKFZ peptide facility | N/A |
| SUMO2/3-epitope peptide  (IRFRFDGQPI) | Peptide synthesis by DKFZ peptide facility | N/A |
| Polyethylenimine, PEI | PolySciences | 23966  Lot # 621405 |
| PEI transfection reagent  (1 mg/ml Polyethylenimine, pH 7.0) | self-made | N/A |
| Hybridoma-SFM | Gibco/Thermo Scientific | 12045076 |
| “SILAC” DMEM (without stable arginine, lysine and glutamine) | Silantes | 280001300  varying Lots |
| 2H-labeled D4-Lysine 2HCl (D4-Lys) | Silantes | 211103913  varying Lots |
| 13C-labeled L-Arginine HCl (^13^C-Arg) | Silantes | 201203902  varying Lots |
| Unlabeled L-Lysine HCl | Silantes | 211004102  varying Lots |
| Unlabeled L-Arginine HCl | Silantes | 201004102  varying Lots |
| G418 disulfate salt | Sigma Aldrich | A1720  varying Lots |

| Critical Commercial Assays | | |
| --- | --- | --- |
| SimpleChIP® Enzymatic Chromatin IP Kit | Cell Signaling Technology | 9003  Lot # 1 |
| Immobilon Western HRP Substrate | Merck Millipore | WBKL50500  varying Lots |
| SuperSignal™ West Pico PLUS | Thermo Scientific | 34580  varying Lots |
| LightCycler® 480 SYBR Green I Kit | Roche | 04707516001  varying Lots |
| Lipofectamine RNAiMAX | Invitrogen | 13778150  varying Lots |
| Deposited Data | | |
| Mass spectrometry data | This paper | PXD018049  Dataset EV1 |
| Microarray data IRF2BP1 WT and IRF2BP1 K597R in full medium, serum starvation and EGF treatment | This paper | GSE135221  Dataset EV2 |
| Microarray data IRF2BP1 and IRF2BP2 knockdown | This paper | GSE161716  Dataset EV3 |
| Experimental Models: Cell Lines | | |
| Hela cells | ATCC | ATCC^®^ CCL-2^™^ |
| Polyclonal Hela cells stably transfected with low expressing pIRES-hrGFPII (“empty vector cells” or “EV cells”) | This paper | N/A |
| Polyclonal Hela cells stably transfected with low expressing IRF2BP1 wildtype, siRNA resistant, in pIRES-hrGFPII (“wildtype cells” or “WT cells”) | This paper | N/A |
| Polyclonal Hela cells stably transfected with low expressing IRF2BP1 K579R, siRNA resistant, in pIRES-hrGFPII (“K579R cells” or “KR cells”) | This paper | N/A |
| Polyclonal Hela cells stably transfected with low expressing IRF2BP1 V578A, siRNA resistant, in pIRES-hrGFPII (“V578A cells” or “VA cells”) | This paper | N/A |
| SUMO1 (21C7) hybridoma cells | Developmental Studies Hybridoma Bank | SUMO-1 21C7-s |
| SUMO2/3 (8A2) hybridoma cells | Developmental Studies Hybridoma Bank | SUMO-2 8A2-s |
| Oligonucleotides (5’🡪3’) | | |
| Primers for cloning HA-IRF2BP1 from Hela cDNA  TCAGGATCCATGGCGTCTGTGCAGGCG  AGTCTCGAGCTAGGGGTCCCGTTCTTTC | This paper | N/A |
| Internal sequencing primers for IRF2BP1  ACGCTGGCACCCGGCTTG  GGAAGATGACCACCGAGG | This paper | N/A |
| Primers for mutagenesis of IRF2BP1 K247R  CGTTCAATGTGCGCTTCAGGAAGGATCACGGGC  GCCCGTGATCCTTCCTGAAGCGCACATTGAACG | This paper | N/A |
| Primers for mutagenesis of IRF2BP1 K579R  GGAGACATCAAAGTTAGGAAAGAACGGGACCCC  GGGGTCCCGTTCTTTCCTAACTTTGATGTCTCC | This paper | N/A |
| Primers for mutagenesis of IRF2BP1 V578A  CTGGAGACATCAAAGCTAAGAAAGAACGGG  CCCGTTCTTTCTTAGCTTTGATGTCTCCAG | This paper | N/A |
| Primers for cloning IRF2BP1-pIRES-hrGFPII from HA-IRF2BP1  AATTGGATCCATGGCGTCTGTGCAGGCGTCCCGCCGCCAGTGGT  TTAAGAATTCCTAGGGGTCCCGTTCTTTCTTAACTTTGATG | This paper | N/A |
| Primers for mutagenesis of IRF2BP1 (siRNA resist.)  GCACTGGCTTCTTCGGGCTTTAAATATCTTGAATATGAACGCCGG  CCGGCGTTCATATTCAAGATATTTAAAGCCCGAAGAAGCCAGTGC | This paper | N/A |
| Primers for cloning HA-IRF2BP2 from Hela cDNA  TCAGGATCCATGGCCGCGGCGGTGGC  AGTCTCGAGTCACGAGTCTCTCTCTTTTTTC | This paper | N/A |
| Internal sequencing primers for IRF2BP2  CCCGTGAACGGCATCCTG  GCCGTGGCTGTCCACATC | This paper | N/A |
| Primers for mutagenesis of IRF2BP2 K326R  GAGAGCAAGTTTAGGAAGGAGCCGGCCC  GGGCCGGCTCCTTCCTAAACTTGCTCTC | This paper | N/A |
| Primers for mutagenesis of IRF2BP2 K566R  GCTGGAGATGTGAAAGTGAGAAAAGAGAGAGACTCG  CGAGTCTCTCTCTTTTCTCACTTTCACATCTCCAGC | This paper | N/A |
| siRNA targeting sequence: IRF2BP1 #5:  GCUUCAAGUACCUCGAAUA[dT][dT]  UAUUCGAGGUACUUGAAGC[dT][dT] | This paper | N/A |
| siRNA targeting sequence: IRF2BP2 #13:  GAGAGAGACUCGUGACUUU[dT][dT]  AAAGUCACGAGUCUCUCUC[dT][dT] | This paper | N/A |
| siRNA targeting sequence: non-targeting:  AAUAGCGACUAAACACAUCAA[dT][dT]  UUGAUGUGUUUAGUCGCUAUU[dT][dT] | This paper | N/A |
| Dusp1 ChIP/qPCR primers “TSS” (-108/+112)  CCCGTCACGTGATCACCATTCAA  GACTGAGAGAGGAGCGTCACGC | This paper | N/A |
| Dusp1 ChIP/qPCR primers “TSS-67” (-243/-67)  AAGAGCAGGCCGGACAGCAG  GGGAGGGGGGGTGTTTGTTTGAATG | This paper | N/A |
| Dusp1 ChIP/qPCR primers “TSS-224” (-473/-224)  GAGTATGCAAAAGCACAGGAAGCCCC  TGCTGTCCGGCCTGCTCTTG | This paper | N/A |
| Dusp1 ChIP/qPCR primers “TSS-961” (-1170/-961)  GGCAATCCGCCCACAATGGC  ACGTTTCTGTCACCTTGGCTCCAGACT | This paper | N/A |
| ATF3 ChIP/qPCR primers “ATF-TSS-241” (-358/-241)  CATTGGTCATGCCTGGAACAC  CCTCGCTCACTGAGACACAC | (Fang *et al*, 2020) | N/A |
| Dusp1 qPCR primers (1606-2112)  CGAGGCCATTGACTTCATAG  CTGGCAGTGGACAAACACC | This paper | N/A |
| ATF3 qPCR primers  AGCCTGGAGCAAAATGATGCTT  AGGTTAGCAAAATCCTCAAACA | This paper | N/A |
| FOS qPCR primers  CTACCACTCACCCGCAGACT  AGGTCCGTGCAGAAGTCCT | This paper | N/A |
| EGR2 pPCR primers  AACGGAGTGGCCGGAGAT  ATGGGAGATCCAACGACCTCTT | (Fang *et al*, 2011) | N/A |
| Actin beta (ACTB) qPCR primers  GACGACATGGAGAAAATCTG  ATGATCTGGGTCATCTTCTC | This paper | N/A |
| Recombinant DNA | | |
| Plasmid: HA-vector  (modified pcDNA3 with an N-terminal HA-tag) | Melchior lab | N/A |
| Plasmid: HA-IRF2BP1 wildtype in pcDNA4 | This paper | N/A |
| Plasmid: HA-IRF2BP1 K247R in pcDNA4 | This paper | N/A |
| Plasmid: HA-IRF2BP1 K579R in pcDNA4 | This paper | N/A |
| Plasmid: HA-IRF2BP2 wildtype in pcDNA4 | This paper | N/A |
| Plasmid: HA-IRF2BP2 K326R in pcDNA4 | This paper | N/A |
| Plasmid: HA-IRF2BP2 K566R in pcDNA4 | This paper | N/A |
| Plasmid: pIRES-hrGFPII | Agilent | 240032 |
| Plasmid: IRF2BP1 wildtype, siRNA resistant, in pIRES-hrGFPII | This paper | N/A |
| Plasmid: IRF2BP1 K579R, siRNA resistant, in pIRES-hrGFPII | This paper | N/A |
| Plasmid: IRF2BP1 V578A, siRNA resistant, in pIRES-hrGFPII | This paper | N/A |
| Software and Algorithms | | |
| The Human Protein Atlas | (Uhlén *et al*, 2005; 2017) | <https://www.proteinatlas.org/> |
| Clustal Omega | (Madeira *et al*, 2019) | <https://www.ebi.ac.uk/Tools/msa/clustalo/> |
| PANTHER | (Mi *et al*, 2019) | <http://www.pantherdb.org/> |
| GSEA | (Subramanian *et al*, 2005) |  |
| GEO (Gene expression omnibus) | (Edgar *et al*, 2002) | <https://www.ncbi.nlm.nih.gov/gds> |
| R Version 3.3.3 |  |  |
| R Package ChIPpeakAnno_3.8.9 |  |  |
| R Package org.Mm.eg.db_3.4.0 |  |  |
| Other | | |
| “Affymetrix” GeneChip™ HuGene 2.0 ST Array Format 100, 6pk | appliedbiosystems | 902112  Lot # 4335317 |
| Falcon® 5 ml Round Bottom Polysyrene Test Tube, with Cell Strainer Snap Cap | Falcon | 352235  varying Lots |
| CELLine classic 1000 | Integra | 90005 |

**References**

Bossis G & Melchior F (2006) Regulation of SUMOylation by reversible oxidation of SUMO conjugating enzymes. *Mol Cell* **21:** 349–357

Edgar R, Domrachev M & Lash AE (2002) Gene Expression Omnibus: NCBI gene expression and hybridization array data repository. *Nucleic Acids Res.* **30:** 207–210

Fang F, Ooka K, Bhattachyya S, Wei J, Wu M, Du P, Lin S, Del Galdo F, Feghali-Bostwick CA & Varga J (2011) The Early Growth Response Gene. *AJPA* **178:** 2077–2090

Fang J, Ji Y-X, Zhang P, Cheng L, Chen Y, Chen J, Su Y, Cheng X, Zhang Y, Li T, Zhu X, Zhang X-J & Wei X (2020) Hepatic IRF2BP2 Mitigates Nonalcoholic Fatty Liver Disease by Directly Repressing the Transcription of ATF3. *Hepatology* **71:** 1592–1608

Hutten S, Flotho A, Melchior F & Kehlenbach RH (2008) The Nup358-RanGAP complex is required for efficient importin alpha/beta-dependent nuclear import. *Mol Biol Cell* **19:** 2300–2310

Madeira F, Park YM, Lee J, Buso N, Gur T, Madhusoodanan N, Basutkar P, Tivey ARN, Potter SC, Finn RD & Lopez R (2019) The EMBL-EBI search and sequence analysis tools APIs in 2019. *Nucleic Acids Res.* **47:** W636–W641

Mi H, Muruganujan A, Huang X, Ebert D, Mills C, Guo X & Thomas PD (2019) Protocol Update for large-scale genome and gene function analysis with the PANTHER classification system (v.14.0). *Nat Protoc* **14:** 703–721

Subramanian A, Tamayo P, Mootha VK, Mukherjee S, Ebert BL, Gillette MA, Paulovich A, Pomeroy SL, Golub TR, Lander ES & Mesirov JP (2005) Gene set enrichment analysis: a knowledge-based approach for interpreting genome-wide expression profiles. *Proc Natl Acad Sci USA* **102:** 15545–15550

Uhlén M, Björling E, Agaton C, Szigyarto CA-K, Amini B, Andersen E, Andersson A-C, Angelidou P, Asplund A, Asplund C, Berglund L, Bergström K, Brumer H, Cerjan D, Ekström M, Elobeid A, Eriksson C, Fagerberg L, Falk R, Fall J, et al (2005) A human protein atlas for normal and cancer tissues based on antibody proteomics. *Mol Cell Proteomics* **4:** 1920–1932

Uhlén M, Zhang C, Lee S, Sjöstedt E, Fagerberg L, Bidkhori G, Benfeitas R, Arif M, Liu Z, Edfors F, Sanli K, Feilitzen von K, Oksvold P, Lundberg E, Hober S, Nilsson P, Mattsson J, Schwenk JM, Brunnström H, Glimelius B, et al (2017) A pathology atlas of the human cancer transcriptome. *Science* **357:** eaan2507
